# Nine Months into the COVID-19 Pandemic: A Longitudinal Study Showing Mental Health and Movement Behaviours Are Impaired in UK Students

**DOI:** 10.3390/ijerph18062930

**Published:** 2021-03-12

**Authors:** Matthew J. Savage, Philip J. Hennis, Daniele Magistro, James Donaldson, Laura C. Healy, Ruth M. James

**Affiliations:** SHAPE Research Group, School of Science and Technology, Nottingham Trent University, Nottingham NG11 8NS, UK; matthew.savage@ntu.ac.uk (M.J.S.); philip.hennis@ntu.ac.uk (P.J.H.); daniele.magistro@ntu.ac.uk (D.M.); james.donaldson@ntu.ac.uk (J.D.); laura.healy@ntu.ac.uk (L.C.H.)

**Keywords:** student, mental health, physical activity, sedentary behaviour, COVID-19, pandemic

## Abstract

Initial studies indicated that student mental health was impaired during the early stages of the pandemic and that maintaining/improving physical activity gave some protection from mental illness. However, as the pandemic persists, these data may not reflect current circumstances and may have been confounded by exam stress. Methods: This study used an online survey to assess the changes in, and associations between, mental health and movement behaviours in 255 UK university students from before the COVID-19 pandemic (October 2019) to 9 months following the UK’s first confirmed case (October 2020). Changes in and associations between mental wellbeing, perceived stress, physical activity, and sedentary behaviour were assessed using a mixed model ANOVA; a multiple linear regression model determined the predictive value of variables associated with Δ mental wellbeing. Results: Mental wellbeing and physical activity decreased (45.2 to 42.3 (*p* < 0.001); 223 to 173 min/week (*p* < 0.001)), whereas perceived stress and time spent sedentary increased (19.8 to 22.8 (*p* < 0.001); 66.0 to 71.2 h/week (*p* = 0.036)). Δ perceived stress, Δ sedentary behaviour and university year accounted for 64.7%, 12.9%, and 10.1% of the variance in Δ mental wellbeing (*p* < 0.001; *p* = 0.006; *p* = 0.035). Conclusion: The COVID-19 pandemic is having a sustained negative impact on student mental health and movement behaviour.

## 1. Introduction

Since the initial outbreak of COVID-19, students have been forced to significantly modify their living and working arrangements and adapt to remote pedagogical practices. These alterations, in conjunction with government enforced restrictions, appear to have substantially impaired university students’ mental health during the early stages of the pandemic [1,2,3,4,5,6,7]. Importantly, maintaining or increasing physical activity levels have previously been shown to be a successful strategy to mitigate reductions in physical and mental health [8,9]. Therefore, it is worrying that a large number of studies have reported reduced physical activity levels and increased sedentary behaviour in university students during the first 2–4 months of the pandemic [7,10,11,12,13,14,15]. Indeed, during the initial phases of the pandemic, several studies indicated that university students who had reduced levels of physical activity tended to have worse mental health [11,16,17], whereas those who maintained or increased their levels of physical activity had better mental health [3,18,19]. Overall, early findings indicate that the mental health of university students has been negatively impacted during the initial stages of the COVID-19 pandemic and that maintaining or increasing physical activity levels may have some protective effect.

In the context of already poor mental health in university students, a further reduction in mental health and movement behaviours in the initial stages of the COVID-19 pandemic is cause for concern. However, the pandemic is an evolving situation in which the UK government are continuously assessing whether restrictions need to be more or less rigorous. At the time of data collection, the UK had entered and exited a three-month lockdown period (23 March 2020–4 July 2020) and was being governed by a tiered framework whereby restrictions were imposed dependent on the localised rate of infection. As such, the negative trends that were observed during the early phase of the pandemic may no longer reflect current trends. Indeed, although the mental health of UK adults was shown to be impaired during the first month of the initial UK ‘lockdown’ [20], recent data suggest that the general population experienced reduced symptoms of anxiety and depression from March 2020 to August 2020 [21]. Therefore, although university students experienced impaired mental health and movement behaviours during the initial stages of the pandemic [7], it is possible that this trend has shifted as the situation persists. Furthermore, given that university students’ mental health is uniquely impacted based on the cycle of the academic year [22], it is plausible that previous cohort studies reporting changes in student mental health due to COVID-19 restrictions may have been confounded by specific factors associated with academia such as exam stress.

The impact of the COVID-19 pandemic will continue to be felt through 2021 and beyond. Given that some degree of nationwide restrictions look set to continue across much of the world for the foreseeable future, it is fundamental that higher education institutions understand the ongoing influence of such restrictions on students’ mental health to inform future policy and practice. Consequently, the present study aims to longitudinally investigate the changes in and associations between mental wellbeing, perceived stress, physical activity and sedentary behaviour in university students from prior to the COVID-19 pandemic (October 2019) to 9 months following the first confirmed case of COVID-19 in the UK (October 2020).

## 2. Materials and Methods

**Figure 1:**
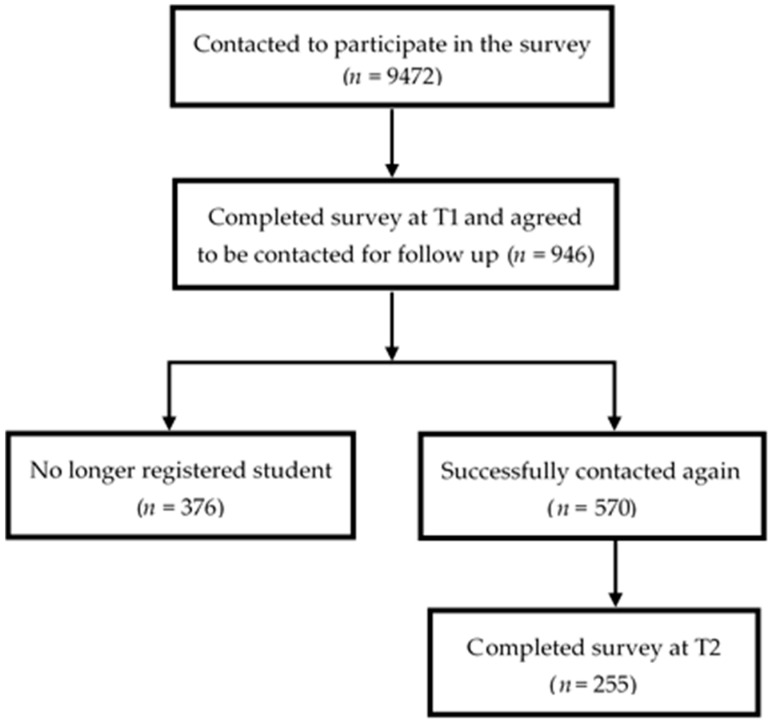
Flow chart displaying the participant recruitment process for the study.

Participants were enrolled in a longitudinal cohort study that investigates the health and well-being of University students in the East Midlands, UK. In term one of the 2019–2020 academic year (14 October 2019–4 November 2019; T1) 9472 students were invited to complete the survey. Of these, 946 responded and also agreed to be contacted again for follow up. Of these, 570 were able to be contacted again in term one of the 2020–2021 academic year (19 October 2020–1 November 2020; T2) and 255 students completed the survey for a second time, thereby forming the cohort analysed in this paper (Figure 1). Participants were informed of the purpose of the study and provided informed consent prior to undertaking the survey. All data were pseudo-anonymised and remained confidential. Ethical approval was granted by the Science and Technology College Research Ethics Committee of the University.

The survey contained socio-demographic questions (8 items; Table 1) and a health history question (Do you suffer from any diagnosed long-term health condition(s)?; 1 item). It contained questions to assess moderate to vigorous physical activity levels (MVPA) using the Exercise Vital Sign (EVS) questionnaire [23] and a question to assess sedentary behaviour [24]. MVPA was calculated by multiplying the number of days per week the participant conducted MVPA by the minutes per day the participant undertook exercise at this level.

The survey contained two scales, the Warwick-Edinburgh Mental Wellbeing Scale (WEMWBS) and the Cohen’s Perceived Stress Scale (PSS) [25]. The WEMWBS uses a 5-point Likert scale (1 = ‘None of the time’ to 5 = ‘All of the time’) where the outcome score ranges from 14 to 70 and higher scores indicate greater mental wellbeing. The PSS also uses a 5-point Likert scale (0 = ‘Never’ to 4 = ‘Very often’) where the outcome score ranges from 0 to 40 and higher scores indicate greater levels of perceived stress. Both the WEMWBS and PSS have previously been validated in a UK student population [26,27].

To determine whether there was a change over time in mental health scores (mental wellbeing and perceived stress) and movement behaviour (sedentary behaviour and MVPA), repeated measures using linear mixed models with “time” as the fixed factor (T1 and T2) were run using the mixed procedure in SPSS v20.0 (IBM. Chicago, IL, USA). The models controlled for the within-subjects nature of the data by including random effects for participant, with a variance component, covariance structure and maximum likelihood estimation. The variation among the timepoints was captured directly by modelling the variance-covariance matrix of the residuals on each timepoint for each participant. The covariance structure for the residuals across timepoints was autoregressive heterogenous, which allowed heterogeneous variances and considered the variables correlated at different timepoints. Additionally, effect size was evaluated using Cohen’s d and the magnitude of change was determined using the following parameters: trivial effect (<0.2) small effect (≥0.2), medium effect (≥0.5), and large effect (≥0.8) [28]. A multiple linear regression model was used to determine which factors were associated with the change in mental wellbeing from October 2019 to October 2020. The analysis used the pre-existing factors assessed before the outbreak and the changes in perceived stress, sedentary behaviour and MVPA. The changes (delta; Δ) were calculated by subtracting T1 scores from T2 scores. For all analysis, significance was accepted at *p* < 0.05.

## 3. Results

### 3.1. Changes in Mental Wellbeing, Stress, Sedentary Behaviour and MVPA

The socio-demographic characteristics of the 255 participants are displayed in Table 1. Participants included in the study were predominantly female (72.7%), white (82.0%), and non-obese (65.9%).

**Figure 2:**
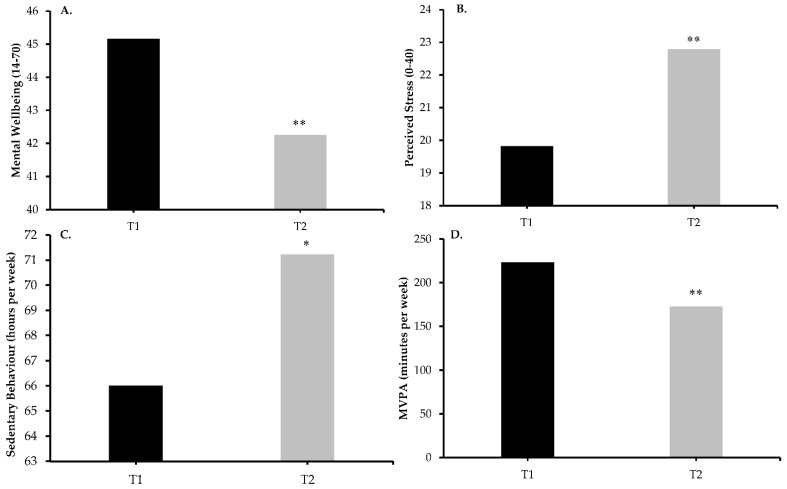
Changes in variables over time. (**A**) Mental wellbeing was lower at T2 compared to T1; (**B**) Perceived stress was greater at T2 compared to T1; (**C**) Sedentary behaviour was greater at T2 compared to T1; (**D**) MVPA was lower at T2 compared to T1. ** indicates *p* < 0.001; * indicates *p* < 0.05.

Changes in mental wellbeing and stress are displayed in Figure 2A,B. Linear mixed model analysis revealed that mental wellbeing was lower at T2 compared to T1 (42.3 vs. 45.2, β = 2.91, t = 5.34, *p* < 0.001) with a small effect size (d = 0.30), and perceived stress was higher at T2 compared to T1 (22.8 vs. 19.8, β = −2.97, t = −6.88, *p* < 0.001), again with a small effect size (d = 0.44). Changes in sedentary behaviour and MVPA are shown in Figure 2C,D. Linear mixed model analysis revealed that sedentary behaviour was greater at T2 compared to T1 (71.2 vs. 66.0 h/week, β = −312.94, t = −2.11, *p* < 0.05) with a trivial effect size (d = 0.16), and MVPA was lower at T2 compared to T1 (173 vs. 223 min/week, β = 50.43, t = 4.81, *p* < 0.001) with a small effect size (d = 0.26). The summary of the three fit-criterion details are available in Table S1.

### 3.2. Factors Associated with Changes in Mental Wellbeing

The multiple regression model is displayed in Table 2. When considering all independent variables, the model was associated with Δ mental wellbeing (R^2^ = 0.429, F(7, 246) = 26.40, *p* < 0.001). Δ perceived stress negatively affected 64.9% of Δ mental wellbeing (*p* < 0.001), Δ sedentary behaviour negatively affected 13.5% of Δ mental wellbeing (*p* < 0.05), and university year group positively affected 10.2% of Δ mental wellbeing (*p* < 0.05). No other variables significantly affected Δ mental wellbeing.

## 4. Discussion

This study investigated the changes in, and associations between, mental wellbeing and movement behaviours in UK university students during the extended period of restrictions resulting from the COVID-19 pandemic. The key findings were that, nine months into the outbreak of COVID-19, mental wellbeing and physical activity were reduced, and perceived stress and sedentary behaviour were increased in university students. Additionally, the increase in perceived stress and sedentary behaviour, and the year group at university, were associated with the decrease in mental wellbeing. Overall, the current study demonstrates that impaired mental health and movement behaviours in university students has been sustained over the initial nine months of the pandemic, and that this decline may be influenced by a multitude of factors arising from prolonged COVID related restrictions.

Initial studies reported that student mental health and movement behaviours were impaired during the initial phases of the pandemic [1,2,3,4,5,6,7,10,11,12,13,14,15], but these studies may no longer be reflective of current trends. Recent findings have indicated that people within the UK general population are becoming accustomed to the new psychological demands of life in lockdown and as a result, mental health is stabilising from high levels of anxiety and depression initially observed during the early stages of the pandemic [21]. However, the findings of the current study demonstrate that mental health has been impaired in university students 9 months following the initial COVID-19 outbreak. Some explanation may be the unique academic, social and financial [29,30] circumstances of students, meaning they are not adapting to government-imposed restrictions in the same way as the general population. Irrespective of the reasons, the clear indication that the COVID-19 pandemic has led to a decline in the mental health of university students should be of great concern to higher education institutions. Previous research has identified cognitive behavioural therapy and mindfulness-based interventions as successful strategies to combat reductions in students’ mental health [31], although it is unknown whether these interventions are still effective within the context of COVID-19. As such, it is vital that higher education institutions, public health systems and researchers work cohesively to develop, implement and evaluate strategies to mitigate the compounding effect of the COVID-19 pandemic on students’ mental health.

The current study highlights a reduction in physical activity and an increase in sedentary behaviour following nine months of the pandemic. This sustained impairment in movement behaviours could have severe consequences for students’ long-term health given that university is a key period in which health-related habits are formed [32]. Indeed, previous studies have identified negative links been between reduced movement behaviours and outcomes of cardiometabolic and mental health in young people [33,34]. Taken together, these findings should be of great concern to public health systems and higher education institutions, particularly against the backdrop of poor health and health-related behaviours reported in university students prior to the pandemic [35,36,37,38]. To mitigate reductions in physical and mental wellbeing during the pandemic, it has been suggested that people undertake home-based exercise [39]. However, the lack of financial resources available to the student population may mean that they are unable to access the relevant equipment to undertake such activities [40]. It is therefore vital that future research addresses the barriers to exercise that students are currently facing, and develops effective interventions in order to attenuate the potential health issues that may arise as a result of the reduction in movement behaviours of university students during the pandemic.

The COVID-19 pandemic is an everchanging situation in which new challenges arise regularly and as such, the factors influencing changes in mental wellbeing may also change. The current study found that the increase in perceived stress, increase in sedentary behaviour, and the year group at university were all related to the decrease in mental wellbeing 9 months into the pandemic. The relationship between perceived stress and aspects of mental health has been well-established in university students and it is therefore unsurprising that this link has remained throughout the COVID-19 pandemic [7,41]. However, in contrast to early findings, the current study found no relationship between physical activity levels and mental wellbeing [3,11,16,17,18,19]. Importantly, this may be driven by the finding that the average MVPA per week in the present population was 173 min, thereby remaining above the recommended guideline of 150 min and not affecting mental wellbeing. However, in contrast, the current study did find that the increase in sedentary behaviour was related to the decrease in mental wellbeing. Previously, increased sedentary behaviour, and specifically screen time, has been linked to poorer mental health in young people [42], and during the initial phase of the pandemic it was reported that university students were engaging in more screen time, largely due to increased social isolation [43]. Consequently, continued restrictions could be further negatively influencing trends associated with sedentary behaviour and screen time, thus negatively affecting mental health. As such, in addition to improving physical activity levels, higher education institutions should aim to develop initiatives that encourage students to reduce their engagement in sedentary activities to further mitigate reductions in mental wellbeing during the pandemic.

The present findings also indicate that university year group is related to the reduction in students’ mental wellbeing nine months into the pandemic. Although the potential mediating mechanisms controlling this are beyond the scope of the current study, it has previously been shown that university students display greater psychological symptoms during their second year of studying due to an increase in academic, social, and financial stressors [44]. It is likely that these stressors remain within the context of COVID-19, and as such are adversely affecting mental health. Furthermore, a recent study has reported that females (irrespective of ethnicity) and men from a Black, Asian, and minority ethnic (BAME) background were experiencing poorer mental health compared to White British men during the initial phase of pandemic [45]. However, the current study found that neither gender nor ethnicity were related to the decline in the mental wellbeing of university students nine months into the pandemic.

A major strength of the current study is the one-year gap between time points (October 2019 to October 2020), enabling the authors to state that the reductions seen in mental health and movement behaviours are not attributable to specific factors associated with academia such as exam stress [22], but are more likely as a result of a multitude of factors associated with the pandemic. These data add to existing literature that have shown acute changes in mental health during the early stages of the pandemic [1,2,3,4,5,6,7,10,11,12,13,14,15,16,17,18,19,46]. Additionally, the duration of the current study has enabled the identification of a sustained reduction in the mental health and movement behaviours of university students nine months into the pandemic. This is further evidenced by the indication that the mental wellbeing and perceived stress of the current population is consistent with that of a similar cohort in April 2020 (42 and 23 vs. 41 and 23, respectively) [7]. Therefore, it appears that the reduction in students’ mental health is an ongoing, long-term issue rather than an initial reaction due to the uncertainty of the situation during the early stages of the pandemic.

The current study uses self-report questionnaires which could have led to inaccuracies in the results, particularly to an overestimation of physical activity [47]. Additionally, the current study did not measure light physical activity levels, which could have increased as compensation for the decline seen in MVPA and which may have implications for mental health. However, whilst increasing light physical activity seems to reduce depression in older adults, whether a similar relationship exists in young people is, as yet, unclear [48]. Furthermore, the lack of multiple assessment points within the current study means that it is not possible to establish when the decline in mental health occurred. However, the majority of previous studies have shown a decline in university students mental health in the early phase the pandemic [1,2,3,4,5,6,7,11,16,17,19,46] and it is therefore likely that this study provides evidence that this reduction has been sustained. The current study did not explore all components of mental health, but changes in mental wellbeing and perceived stress were assessed using scales validated in the current population [26,27]. Finally, the nature of survey-based research meant that the response rate to the current survey was greater in females compared to males and the attrition rate was high from T1 to T2. However, these are well-known limitations of survey-based research in young adults [49,50,51,52], and there was no influence of gender in the regression model. Despite these limitations, the findings of the current study clearly indicate that, nine months into the pandemic, there is a sustained reduction in mental wellbeing in university students, and perceived stress, sedentary behaviour and the year group at university are partly responsible.

## 5. Conclusions

The current study found that, nine months after the initial outbreak of COVID-19 in the UK, the mental health and movement behaviours of university students has been impaired. The decline in mental wellbeing was related to increased perceived stress, increased sedentary behaviour, and the year group at university. Overall, these results demonstrate that the pandemic is having a sustained negative influence on students’ mental health and movement behaviours, which may be influenced by a multitude of factors arising from prolonged lifestyle restrictions. Given that these restrictions are set to continue for some time, it is imperative that universities and public health systems consider these results and develop long-term strategies to mitigate further declines in student mental health.

## Figures and Tables

**Table 1:** Participant information.

	N (%) or Mean ± SD
**Age (y)**	
18	1 (0.4)
19	40 (15.7)
20	72 (28.2)
21	65 (25.5)
22–25	51 (20.0)
26–35	20 (7.8)
35+	6 (2.4)
**Gender**	
Male	59 (23.1)
Female	193 (75.7)
Neither of the above/other	2 (0.8)
Prefer not to say	1 (0.34)
**Ethnicity**	
White	209 (82.0)
Mixed	9 (3.5)
Asian	23 (9.0)
Black	9 (3.5)
Other	3 (1.2)
Prefer not to say	2 (0.8)
**Height (m)**	1.68 ± 0.10
**Weight (kg)**	70.2 ± 18.1
**BMI**	24.9 ± 6.0
**University year group**	
Year 1	3 (1.2)
Year 2	88 (34.5)
Year 3	108 (42.34)
Year 4	36 (14.1)
Other/Not answered	20 (7.8)
**Diagnosed long-term mental health condition**	
No mental health condition	178 (69.8)
Any mental health condition	77 (30.2)
Anxiety (Singular)	19 (24.7)
Depression (Singular)	4 (5.2)
Anxiety and Depression	36 (46.8)
OCD	5 (6.5)
Mood disorder	5 (6.5)
Eating disorder	3 (3.9)
Insomnia	2 (2.6)
PTSD	2 (2.6)
Schizophrenia	1 (1.3)

BMI = body mass index; SD = standard deviation; OCD = obsessive compulsive disorder; PTSD = post-traumatic stress disorder.

**Table 2:** Multiple regression model displaying factors associated with Δ mental wellbeing.

	Unstandardized Coefficients		Standardized Coefficients	t	*p*
	B	SE	Beta		
Gender	1.453	0.871	0.078	1.668	0.097
Ethnicity	0.098	0.098	0.048	0.997	0.320
University year group	0.978	0.461	0.102	2.125	0.035
Δ Perceived stress	−0.817	0.061	−0.649	−13.468	<0.001
Δ Sedentary behaviour	−0.000	0.000	−0.135	−2.761	0.006
Δ MVPA	0.000	0.003	0.005	0.108	0.914

## Data Availability

Requests for all data should be submitted to the corresponding author for review. Following consideration, access to anonymised data may be approved.

## Supplementary Materials

The following are available online at https://www.mdpi.com/1660-4601/18/6/2930/s1, Table S1: Results of Information Criterion of the Covariance Structure Autoregressive heterogenous for each analysis.
