# Supplementary material for: Nine Months into the COVID-19 Pandemic: A Longitudinal Study Showing Mental Health and Movement Behaviours Are Impaired in UK Students
Source: Int J Environ Res Public Health. 2021 Mar 12;18(6):2930. doi: 10.3390/ijerph18062930 (PMC7999965; doi:10.3390/ijerph18062930)
Supplement: Supplementary file 1 [file ijerph-18-02930-s001.pdf]

Table S1. Results of Information Criterion of the Covariance Structure Autoregressive heterogenous for each analysis.

	-2LL	AIC	BIC
Mental wellbeing	3648.39	3658.39	3679.56
Perceived stress	3333.04	3343.04	3364.22
Sedentary behavior	9155.26	9165.26	9186.43
MVPA	6669.72	6669.72	6700.89

The table summarises the three fit-criterion (-2 times the log-likelihood [-2LL], Akaike's Information Criterion [AIC], and the Bayesian Information Criterion [BIC]) for each analysis.
